# Crisis checklist (Code Red) for the management of cardiac arrest during minimally invasive thoracic surgery: case report

**DOI:** 10.1186/s13019-020-01200-4

**Published:** 2020-07-16

**Authors:** Philippe Rinieri, Jean Selim, Vincent Le Guillou, Jean-Marc Baste

**Affiliations:** 1 Department of General and Thoracic Surgery, Rouen University Hospital, Charles Nicolle Hospital, 1 rue de Germont, 76031 Rouen, France; 2 Department of Anaesthesiology and Intensive Care, Rouen University Hospital, Rouen, France; 3 Department of Cardiac Surgery, Rouen University Hospital, Rouen, France

**Keywords:** Minimally invasive thoracic surgery, Cardiac arrest, Crisis check list, Crisis resource management, Extra corporeal membrane oxygenation, Case report

## Abstract

**Background:**

The management of cardiac arrest during video assisted thoracic surgery is challenging. Checklist use improve the management of operating-room crises.

Case presentation: Cardiac arrest (asystole) occurred during anatomical pulmonary resection by minimally invasive surgery. Conversion to thoracotomy was decided (thoracic surgeon and anesthesiologist conjointly) to check for absence of cardiac bleeding and to start cardiac massage (4 min no-flow). After few minutes, ventricular fibrillation occurred and persisted despite shocks. Extracorporeal life support with veno-arterial extracorporeal membrane oxygenation allowed a return of spontaneous circulation (45 min low-flow).

**Conclusions:**

The patient survived without central neurologic deficit due to perfect team work process using a crisis check-list (strengthened by a comprehensive simulation program with crisis resource management).

## Background

A multicenter series showed a prevalence of 1.5% of major intraoperative complications after video assisted thoracic surgery (VATS) anatomical lung resections [1]. In a high-fidelity simulation study, checklist use was associated with significant improvement in the management of operating-room crises [2]. The aim of this case report is to describe our management of a cardiac arrest (asystole) during VATS segmentectomy and the application of an intraoperative crisis checklist known as “Code Red”. Should we do an internal or external cardiac massage in case of asystole during a VATS procedure? The decision must be very fast and training can help in these emergency situations.

## Case presentation

A 56-year-old man had smoking history at 40 packs / year, no other history. A suspicious pulmonary nodule was detected in the left lower lobe. FEV1 and DLCO values were respectively 93 and 55%. A basal segmentectomy was planned for this increasingly central nodule according to multidisciplinary meeting.

**Fig. 1:**
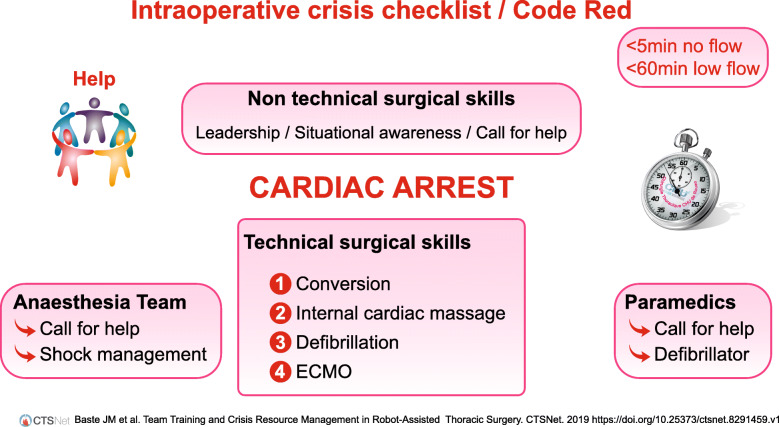
Intraoperative Crisis Checklist / Code Red / Cardiac Arrest

**Fig. 2:**
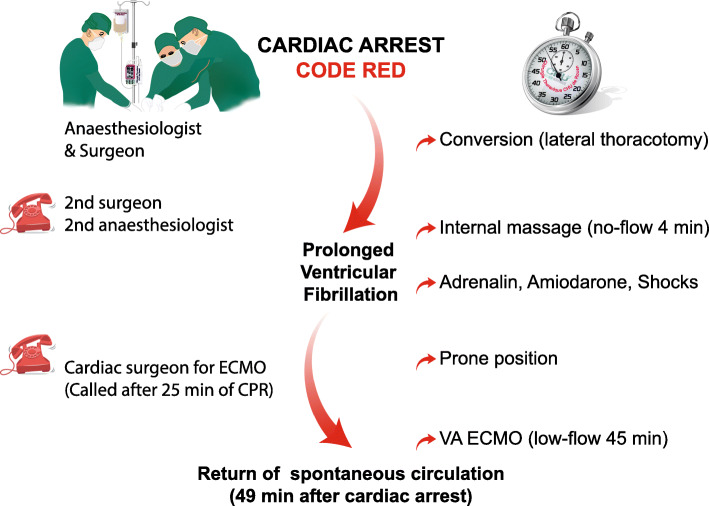
Operatives Times

VATS segmentectomy was started according to the World Health Organisation surgical safety checklist. Cardiac arrest (asystole) occurred immediately after placement of the first port (anterior axillary line, 7th intercostal space). After communication between the thoracic surgeon and anesthesiologist, it was decided to apply the Code Red procedure (Fig. 1), to convert to thoracotomy to check for absence of cardiac bleeding (or pericardial wound) and to start cardiac resuscitation. A second thoracic surgeon and a second anaesthesiologist were immediately called in accordance with the crisis checklist. Internal cardiac massage was started after 4 min no-flow. Cardio pulmonary resuscitation (CPR) was performed according to the guidelines of the European Resuscitation Council guidelines [3]. After few minutes, ventricular fibrillation (VF) occurred. The patient received adrenaline, amiodarone and internal electric shocks during CPR. After 25 min of CPR without cardiac recuperation (persistant VF), the surgical team (thoracic surgeon and anesthesiologist) conjointly decided to contact the cardiac surgeon for a veno-arterial extra corporeal membrane oxygenation (VA ECMO). While the first thoracic surgeon continued the internal cardiac massage, we placed the patient in decubitus position and the second thoracic surgeon surgically prepared the right femoral vessels. The cardiac surgeon placed the peripheral VA ECMO cannula after 45 min low-flow. This allowed a return of spontaneous circulation. Operative times are available in Fig. 2.

In post-operative, coronarography showed coronary disease, without stenting. Then, redo for pericardial hemostasis was done by thoracotomy during the night. Therapeutic hypothermia (36 degrees Celsius) was done over a period of 24 h. The patient was weaned off the pump at Day 2 because of cardiac recovery, then extubated at Day 4 without central neurologic deficit. He was discharged from intensive care unit at Day 7. The left ventricular ejection fraction was 45%. The patient was discharged from hospital at Day 18. Another coronarography with interventricular artery stenting was done at 2 months.

## Discussion and conclusions

In prolonged cardiac arrest with failing conventional measures, rescue by extracorporeal support provides an ultimate therapeutic option [4]. VA ECMO must be decided quickly to treat prolonged cardiac arrest. In our case, we concluded myocardial infarction after induction responsible for asystole. Despite 4 min no-flow and 45 min low-flow, the patient survived without central neurologic deficit, probably thanks to general anesthesia, quick internal cardiac massage, VA ECMO and hypothermia, but mainly due to perfect team cooperation respecting guidelines and checklist.

An in situ intraoperative crisis simulation model for thoracic surgical emergencies was created, implemented, and demonstrated to be effective as a proof of concept at identifying latent threats to patient safety and differentiating the nontechnical skills of trainees and consultant surgeons [5]. We have developed a comprehensive in situ intraoperative crisis simulation program for our thoracic surgery team. The objective of this simulation program is to learn how to use a crisis checklist (respect guidelines and call for help) because we believe in improved operative safety and team work. Our experience allowed us to immediately convert (instruments prepared), call a second thoracic surgeon (identified by name during preoperative checklist), prepare the defibrillator and improve multidisciplinary collaboration. After a brief exchange with the anesthesiologist (situational awareness), the thoracic surgeon decided to convert to thoracotomy to check for absence of cardiac bleeding (port around the heart) and to start CPR.

Our perfect team work process was strengthened by a comprehensive simulation program focussing on human factors and surgical crisis resource management. Our intraoperative crisis simulation program allowed us to apply perfectly our Code Red for the management of cardiac arrest during minimally invasive thoracic surgery.

## Data Availability

Not applicable.

## Abbreviations

VATS: Video assisted thoracic surgery
VA ECMO: Veno-arterial extra corporeal membrane oxygenation
CPR: Cardio pulmonary resuscitation
VF: Ventricular fibrillation
